# Hypofibrinolysis in diabetes: a therapeutic target for the reduction of cardiovascular risk

**DOI:** 10.1186/s12933-017-0515-9

**Published:** 2017-03-09

**Authors:** Katherine Kearney, Darren Tomlinson, Kerrie Smith, Ramzi Ajjan

**Affiliations:** 1 Division of Cardiovascular & Diabetes Research, Leeds Institute of Cardiovascular and Metabolic Medicine (LICAMM), University of Leeds, Leeds, LS2 9JT UK; 2 Biomedical Health Research Centre, Astbury Building, University of Leeds, Leeds, LS2 9JT UK

**Keywords:** Fibrinolysis, Diabetes, Fibrinogen

## Abstract

An enhanced thrombotic environment and premature atherosclerosis are key factors for the increased cardiovascular risk in diabetes. The occlusive vascular thrombus, formed secondary to interactions between platelets and coagulation proteins, is composed of a skeleton of fibrin fibres with cellular elements embedded in this network. Diabetes is characterised by quantitative and qualitative changes in coagulation proteins, which collectively increase resistance to fibrinolysis, consequently augmenting thrombosis risk. Current long-term therapies to prevent arterial occlusion in diabetes are focussed on anti-platelet agents, a strategy that fails to address the contribution of coagulation proteins to the enhanced thrombotic milieu. Moreover, antiplatelet treatment is associated with bleeding complications, particularly with newer agents and more aggressive combination therapies, questioning the safety of this approach. Therefore, to safely control thrombosis risk in diabetes, an alternative approach is required with the fibrin network representing a credible therapeutic target. In the current review, we address diabetes-specific mechanistic pathways responsible for hypofibrinolysis including the role of clot structure, defects in the fibrinolytic system and increased incorporation of anti-fibrinolytic proteins into the clot. Future anti-thrombotic therapeutic options are discussed with special emphasis on the potential advantages of modulating incorporation of the anti-fibrinolytic proteins into fibrin networks. This latter approach carries theoretical advantages, including specificity for diabetes, ability to target a particular protein with a possible favourable risk of bleeding. The development of alternative treatment strategies to better control residual thrombosis risk in diabetes will help to reduce vascular events, which remain the main cause of mortality in this condition.

## Background

Diabetes is becoming the epidemic of the twenty first century with more than 1.5 million deaths worldwide directly attributed to this condition in 2012 (http://www.who.int). Despite advances in therapy, cardiovascular disease (CVD) remains the main cause of morbidity and mortality in individuals with diabetes. The risk of vascular complications is doubled in diabetes, resulting in significant reduction in life expectancy [1, 2]. Moreover, following a vascular event, the outcome in patients with diabetes is worse compared with individuals with normal glucose metabolism, regardless of the therapeutic strategy used in the acute stage [3–7]. There are two key reasons for the adverse vascular outcome in patients with diabetes. The first is related to more extensive vascular pathology and the second involves an enhanced thrombotic environment [8]. There are two main types of diabetes, one is characterised by insulin deficiency, termed type 1 diabetes (T1DM), and the other, type 2 diabetes (T2DM), arising mainly due to insulin resistance secondary to increased prevalence of obesity. However, the two conditions can overlap as a significant proportion of individuals with T1DM develop a phenotype seen in T2DM, making them fall into a new category termed double diabetes [9]. Equally, longer duration of T2DM can lead to insulin deficiency, making these individuals similar to T1DM patients.

Blood clot formation occurs secondary to interactions between the cellular and protein arms of coagulation, resulting in a network of fibrin fibres populated by various blood cells. Diabetes is characterised by enhanced activation of platelets as well as the formation of compact fibrin networks that are resistant to fibrinolysis [10–15], consequently increasing thrombosis risk.

Although the presence of a prothrombotic environment in diabetes is well documented, current anti-thrombotic treatment strategies aiming to reduce vascular risk are largely similar in those with and without diabetes. Moreover, therapies are mainly directed to control platelet activation and the fibrin network is not usually targeted unless there are additional pathologies such as atrial fibrillation or valvular heart disease. Therefore, in order to reduce residual vascular risk in diabetes, more effective anti-thrombotic treatment strategies are required.

This review will summarise current knowledge in fibrin network abnormalities in diabetes with special emphasis on fibrinolysis. The work will also explore diabetes-specific novel anti-thrombotic therapeutic targets aiming to reduce the unacceptably high risk of vascular disease in individuals with diabetes.

## Clot formation and lysis

The formation of a fibrin clot is the final step in the atherothrombotic process, involving complex interactions between platelets and plasma coagulation proteins. After rupture of an atherosclerotic plaque, platelets adhere to the site of injury and become partially activated. Exposed tissue factor (TF) binds factor VII (FVII), promoting proteolysis and activation to FVIIa. The TF/FVIIa complex activates FIX and FX resulting in the generation of FIXa and FXa, and the latter subsequently associates with cofactor FVa to form a prothrombinase complex, which converts prothrombin into thrombin [16]. The generated thrombin converts soluble fibrinogen into insoluble fibrin fibres. Thrombin also activates FXIII, a transglutaminase that crosslinks neighbouring fibrin fibres resulting in the formation of a branched fibrin clot structure, which is more resistant to lysis [17]. Activated FXIII (FXIIIa) crosslinks other proteins into the fibrin network, including plasmin inhibitor (PI) [18], thrombin activatable fibrinolysis inhibitor (TAFI) [19] and plasminogen activator inhibitor-2 (PAI-2) [20], further increasing resistance of the clot to lysis. The coagulation cascade results in the formation of a fibrin mesh, with embedded erythrocytes and other cellular blood elements, which can occlude the vascular lumen, ultimately resulting in the acute complications of CVD, including myocardial infarction, stroke and critical limb ischaemia.

Normal physiology ensures a balance between fibrin clot formation and lysis in order to prevent widespread vascular occlusion or excessive bleeding. Plasmin is the enzyme responsible for fibrin breakdown (fibrinolysis) and is generated from plasminogen through the action of the serine protease tissue plasminogen activator (tPA), a product of endothelial cells [21]. The binding of tPA to fibrin increases the catalytic conversion of plasminogen to plasmin, while also localising the generation of plasmin to the site of thrombus formation. Plasmin cleavage of fibrin generates new C-terminal lysine residues (additional tPA/plasminogen binding sites) within the fibrin network [22].

**Fig. 1:**
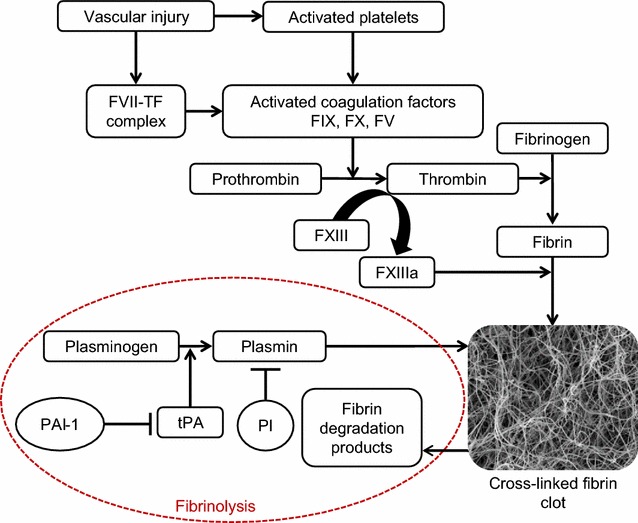
Fibrin clot formation and lysis. Following vascular injury, tissue factor (TF) is released followed by a complex interaction between the cellular and protein arms of coagulation, resulting in activation of various coagulation factors, including FV, FVII, FIX and FX, culminating in the formation of thrombin, which converts soluble fibrinogen into a network of insoluble fibrin fibres. Thrombin also activates factor XIII (FXIII) to active FXIII (FXIIIa), which crosslinks the fibrin fibres and also incorporates antifibrinolytic proteins into the clot. Plasmin, derived from plasminogen by the action of tissue plasminogen activator (tPA), is the enzyme responsible for fibrin clot lysis, resulting in the generation of fibrin degradation products. The main inhibitors of fibrinolysis are plasmin inhibitor (PI) and plasminogen activator inhibitor (PAI-1)

Several proteins prevent unregulated plasmin or plasminogen activator activity, thereby inhibiting excessive clot lysis. Plasminogen activator inhibitor (PAI-1) is produced by endothelial cells, platelets and adipose tissue. Like other serpins (serine protease inhibitors), PAI-1 forms a stable 1:1 complex with tPA, thereby inhibiting its action [23]. Plasmin inhibitor (PI), another member of the serpin family, is the primary physiological inhibitor of plasmin. PI forms a stable inactive complex with plasmin, and can also be covalently cross-linked to fibrin making fibrin more resistant to lysis [24, 25]. Importantly, the fibrinolytic inhibitory properties of PI are significantly enhanced once the protein is cross-linked into the fibrin network. Thrombin activatable fibrinolysis inhibitor (TAFI) cleaves C-terminal lysine residues from partially degraded fibrin, decreasing the number of available plasminogen binding sites [26]. The main factors involved in clot formation and lysis are summarised in Fig. 1. Altered level and/or activity of these antifibrinolytic factors can modulate plasma clot lysis, and thereby thrombosis risk. Several studies have shown an association between hypofibrinolysis and increased thrombosis risk [27–30].

## Factors involved in increased vascular risk in diabetes

**Fig. 2:**
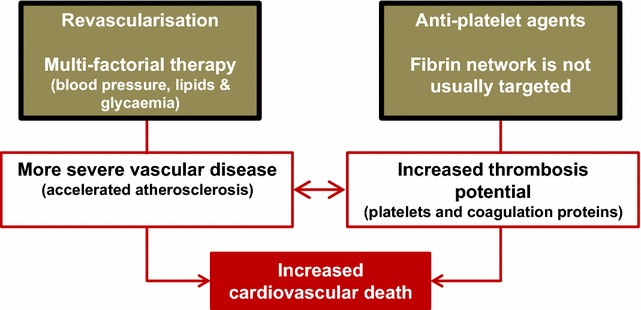
Mechanisms for increased risk of vascular occlusive events in diabetes. Individuals with diabetes have premature atherosclerosis, which increases the risk of a coronary event. Individuals with coronary artery occlusion are treated with revascularisation in the acute stage followed by multifactorial therapy to halt the atherosclerotic process including control of blood pressure, blood glucose and lipid levels. Diabetes is also associated with an enhanced thrombotic milieu, secondary to increased activation of both platelets and coagulation factors. This is treated with anti-platelet therapy and the protein arm of coagulation is not usually targeted except in those with arrhythmias, valvular heart disease or a history of venous occlusion

Individuals with diabetes suffer from premature atherosclerosis, a key contributing factor to increased cardiovascular risk in this population. This is treated in the acute stage with revascularisation followed by preventative therapy in the long-term, including control of blood pressure, lipid and glucose levels. To control thrombosis risk following an acute vascular event, dual antiplatelet therapy is usually used [31, 32], whereas treatment targeting the fibrin network is not considered except in cases of cardiac arrhythmias, valvular heart disease or history of venous thromboembolism. Factors leading to increased vascular events in diabetes and current treatment modalities are summarised in Fig. 2.

The risk of aggressive anti-thrombotic therapy is increased bleeding which may negate any beneficial effect of such therapy. A prime example is the TRITON study that has shown that the combination of prasugrel and aspirin is superior to clopidogrel and aspirin at reducing vascular ischaemia following myocardial infarction but there was an increased risk of bleeding with the former combination making widespread clinical use problematic [33]. Interestingly, patients with diabetes showed a benefit with prasugrel/aspirin therapy without increased risk of bleeding suggesting altered response to anti-thrombotic therapy in this population [32, 34].

## Current anti-thrombotic strategies: pitfalls

Reducing the thrombotic environment in arterial occlusive disease relies mainly on antiplatelet therapy, using the cyclooxygenase inhibitor aspirin with or without P2Y12 antagonists clopidogrel, prasugrel or ticagrelor. While all anti-platelet agents are likely to alter clot structure through indirect effects on platelet-dependent thrombin formation, aspirin is unique amongst anti-platelet agents as it directly alters clot structure and enhances fibrinolysis, related, at least in part, to acetylation of fibrinogen. This makes aspirin an agent that independently targets both the cellular and protein phase of coagulation [35–37]. It remains unclear, however, whether the pro-fibrinolytic effects of aspirin are important clinically and this remains an area for future research. It should be noted that other agents used in diabetes such as hypoglycaemia therapies, anti-hypertensive agents and statins may affect the thrombotic milieu, which is reviewed elsewhere and is not the focus of the current review [38].

Antithrombotic therapy in diabetes can be divided into primary prevention, which includes individuals without a previous history of vascular ischaemia, and secondary prevention in those who have had a vascular occlusive event. Until relatively recently, aspirin has been used for primary vascular protection in diabetes but a number of studies suggest a lack of significant success with such an approach [39]. Therefore, guidelines generally advocate aspirin for primary vascular protection in subgroups of diabetes patients at a higher vascular risk, without clearly identifying these subgroups [8]. Until data becomes available from large primary prevention studies in diabetes, such as ASCEND (NCT00135226), routine aspirin therapy for primary vascular protection in diabetes should be avoided and limited to individuals with multiple risk factors [40].

In individuals sustaining a coronary event, current guidelines advocate the use of dual antiplatelet therapy (DAT) to reduce thrombosis risk for a period of 1 year, followed by lifelong antiplatelet monotherapy [8]. Interestingly, there is generally no differentiation between individuals with and without diabetes when it comes to DAT, despite the documented increased thrombotic environment in those with impaired glucose metabolism. Limited evidence suggests that prasugrel is more effective than clopidogrel at reducing further atherothrombosis in those with diabetes, however ticagrelor appears to have the best risk/benefit profile in those with and without diabetes, and therefore many centres use this agent for all patients regardless of glycaemic status [6, 41].

One year after the vascular event, P2Y12 inhibitor therapy is usually stopped and patients continue on aspirin treatment. However, this approach is questionable in diabetes as the efficacy of aspirin appears to be compromised, secondary to a number of factors including increased platelet protein glycation and higher platelet turnover in diabetes [42]. Improving glycaemia may address the first point but the latter can only be overcome by more frequent aspirin dosing. Indeed, a number of studies have shown a benefit in twice daily aspirin administration on platelet function profile, although outcome studies employing this dosing regimen are lacking [43–45].

One striking observation of clinical studies aiming to reduce atherothrombosis risk is the concentration on antiplatelet therapy, generally ignoring the contribution of the protein arm of coagulation to the vascular ischaemic event. Early work has shown that warfarin, an agent that inhibits the production of various coagulation proteins, can indeed improve outcome after arterial occlusion. However this agent is associated with a narrow safe therapeutic window and the combination with antiplatelet agents greatly increases bleeding risk [46]. More modern agents that affect coagulation proteins, such as FX inhibitor, show a greater promise although their use is still limited by the high bleeding risk when used with DAT [47].

FXII inhibitors offer a potential alternative antithrombotic strategy in diabetes that do not carry the bleeding risk associated with the aforementioned agents. FXII initiates the intrinsic coagulation pathway, however, FXII deficiency in mice is not associated with increased bleeding and offers protection against thrombosis. FXII inhibition may therefore be useful in instances involving activation of the intrinsic pathway (such as stent thrombosis) but may not be appropriate if the underlying pathology is plaque rupture and future research in this area is certainly needed [48].

It should be remembered that acute fibrinolytic therapy has been successfully employed as a treatment for acute vascular occlusion and therefore strategies that facilitate clot lysis on a long-term basis have the potential to control the risk of atherothrombosis, particularly in conditions characterised by impaired fibrinolysis.

Although the current review concentrates on macrovascular complications in diabetes, studies suggest that hypofibrinolysis is also associated with microvascular complications [49–51]. Therefore, addressing the hypofibrinolytic environment in diabetes may help not only by ameliorating macrovascular complications but also by reducing microvascular disease.

Factors involved in increased thrombosis risk in diabetes and current anti-thrombotic strategies are summarised in Table 1.

**Table 1:** Current therapeutic interventions to reduce thrombosis risk in diabetes with their main limitations

Risk factor	Cause	Current therapeutic intervention	Pitfalls of current therapeutic strategy
Premature atherosclerosis	Metabolic abnormalities Inflammatory changes resulting in endothelial dysfunction	Treated in the acute stage with revascularisation Control of blood pressure, lipid and glucose levels in the long term	Mainly preventative Limited ability to reverse vascular pathological changes
Prothrombotic environment	Enhanced platelet activity Altered levels of coagulation factors	Dual antiplatelet therapy (DAT) for 1 year following an acute coronary event Lifelong platelet monotherapy after 1 year	Aspirin resistance in individuals with diabetes Bleeding risk No targeting of the fibrin network
Hypofibrinolysis	Formation of more compact clots Impaired fibrinolytic system	Not usually targeted Agents that modulate the coagulation cascade, which are likely to affect fibrinolysis, are used in the presence of cardiac arrhythmias, valvular heart disease or venous thrombosis	Adding an agent that targets the fibrin network, particularly on a background of DAT, increases the risk of bleeding complications

## Mechanisms of hypofibrinolysis

Hypofibrinolysis is a key abnormality in individuals with diabetes and therefore modulation of this pathological process may offer therapeutic benefits. The main factors influencing fibrinolysis in diabetes include altered fibrin network structure, increased incorporation of antifibrinolytic proteins into the clot and compromised activity of the fibrinolytic system.

### Clot structure in diabetes and factors contributing to altered clot phenotype

Compact fibrin networks with densely packed thin fibres are associated with increased risk of cardiovascular events [52–57], which may be due to reduced permeation of fibrinolytic enzymes into clots with these structures [58]. Although single thick fibrin fibres are cleaved at a slower rate than thin fibres, clots made from an increased number of more densely packed thin fibres are slower to lyse [59]. Additionally, thinner fibrin fibres support a slower rate of tPA-mediated plasmin generation than thick fibres, and a slower rate of fibrin digestion by plasmin [60, 61]. It should be noted that increased incorporation of antifibrinolytic proteins into clots with compact structure further contributes to their resistance to breakdown and therefore the relationship between structure and lysis is more complex than initially envisaged [62].

Individuals with type 1 and type 2 diabetes have prothrombotic compact fibrin networks which correlate with glycaemic control measured as HbA1c [63–66]. To complicate matters, glycaemia-independent factors, such as gender, can also determine fibrin clot structure with female patients exhibiting denser fibrin clots and prolonged lysis time [67]. This may be one of the mechanisms contributing to the loss of cardiovascular protection in women who develop diabetes. Prolonged duration of T2DM (>5 years) is further associated with hypofibrinolysis and a prothrombotic clot phenotype, even if glycaemic control is adequate, adding yet more complexity to studying fibrin network characteristics in this population [68]. These examples emphasise the heterogeneity in thrombosis potential in diabetes and highlight some of the challenges faced in developing safe and effective anti-thrombotic agents in this condition.

#### Mechanisms for altered clot structure in diabetes

A number of mechanisms are responsible for altered clot structure in diabetes including quantitative and qualitative changes in coagulation factors as well as altered thrombin generation.

##### Plasma fibrinogen levels

Previous studies have observed a relationship between elevated plasma levels of fibrinogen and the risk of CVD [69–72]. Evidence suggests that high plasma fibrinogen levels influence clot structure, by modulating network density and rigidity through increasing fibre number and branch points. The increased risk of MI associated with elevated plasma fibrinogen levels may be attributed, in part, to the formation of stiffer clots in these individuals [73]. In vivo work has shown that elevated fibrinogen levels reduce time to vascular occlusion, and increase clot fibrin content, network density and resistance to fibrinolysis in a murine model [74], linking changes in fibrin network to increased risk of thrombosis.

Plasma levels of fibrinogen in diabetes subjects has been a focus of much research, however, results are not always in agreement. The majority of, but not all, studies found elevated plasma fibrinogen levels in individuals with T2DM, and some reported higher levels in individuals with T1DM (summarised in [38]). The Rotterdam study found no significant difference in fibrinogen levels between individuals with T2DM and those with normal glucose metabolism after adjusting for age. However, plasma fibrinogen was significantly elevated in insulin-treated T2DM patients, which may be due to poorer metabolic control in these individuals or longer disease duration [75].

Taken together, elevated fibrinogen levels appear to contribute to the compact fibrin networks observed in diabetes, at least in some patients.

##### Fibrinogen glycation

Protein glycation is a post translational modification, the extent of which is determined by ambient glucose levels and the duration of protein exposure. Glucose is able to bind non-enzymatically to fibrinogen by condensation of the carbonyl groups with amino groups on the fibrinogen molecule. Elevated glucose levels increase plasma protein glycation, including that of fibrinogen [76–78], with a number of lysine residues becoming glycated [79]. This may contribute to more compact clots in diabetes, which in turn increases resistance to fibrinolysis [80, 81]. Moreover, as lysine is involved in the binding of tPA and plasminogen to fibrin, it is possible that fibrinogen glycation reduces binding of fibrinolytic proteins further impairing clot lysis [81]. In support of this hypothesis, Dunn et al. observed a reduction in plasmin generation in individuals with diabetes, attributed to reduced tPA and plasminogen binding to fibrin [65].

Fibrinogen glycation in subjects with diabetes is modulated by improving glycaemic control [76, 78, 82]. Pieters et al. observed that improving glycaemia had no effect on the structure of fibrin clots made from T2DM plasma although an effect on clots made from purified fibrinogen was observed [80, 83]. The discrepancy between plasma and purified systems can be explained by the heterogeneity observed in plasma of patients with T2DM and the relatively small number of samples analysed.

It should be noted, however, that glycation is not the only post-translational modification of fibrinogen in diabetes [63]. Diabetes is also associated with elevated levels of reactive oxygen species [84], which are able to covalently modify protein structure, including that of fibrinogen [85]. Elevated levels of oxidative stress markers have been documented in T2DM, levels of which were inversely correlated with clot permeability and positively correlated with clot lysis time [86]. Therefore, both glycation and oxidation influence fibrin network properties in diabetes.

##### Thrombin generation

Fibrin fibre diameter decreases with increasing thrombin levels. Low thrombin concentrations (<1 nM, <0.1 U/ml) produce clots composed of loosely woven, thick fibres, whilst clots formed at high thrombin tend to be made up of thin, tightly packed fibrin strands which are relatively resistant to lysis [87, 88]. The concentration of thrombin observed during a coagulation reaction ranges from <1 to >500 nM [89], although low levels of thrombin (~2 nM) are sufficient for fibrin polymerisation [90].

Elevated thrombin generation has been reported in subjects with T2DM [68, 91–93]. In acute coronary syndrome, hyperglycaemia is associated with enhanced thrombin generation at the site of vascular injury and unfavourably altered clot features in patients with and without a history of diabetes [94]. Indeed, controlling glucose levels results in reduced thrombin generation [95]. On the other hand, over treatment of hyperglycaemia and precipitation of hypoglycaemia is associated with enhanced thrombin formation and the formation of more dense fibrin clots with resistance to lysis [96]. This indicates that both hyper and hypoglycaemia are prothrombotic and caution should be exercised to avoid low blood glucose with hypoglycaemic therapy.

The mechanisms for enhanced thrombin generation in individuals with diabetes are not entirely clear. A study involving T1DM and T2DM patients showed that high levels of coagulation factors II,V,VII,VIII and X, coupled with low levels of anticoagulation factor protein C in diabetes are likely to be factors contributing to enhanced thrombin generation [91]. In another study, a modest elevation in thrombin generation in individuals with T2DM as well as those with impaired glucose tolerance was observed. In these individuals, central adiposity and related low grade inflammation, rather than glucose levels per se, were the likely explanations for enhanced thrombin generation [97].

##### Interaction between inflammatory and thrombotic proteins

It is well established that low grade inflammation predisposes to atherothrombosis and a number of pathways have been implicated describing interactions between inflammatory and thrombotic molecules, reviewed elsewhere [98–100]. For example, cytokines, important mediators of inflammation, can directly affect thrombosis risk by creating a hypercoagulable milieu and enhancing platelet reactivity [101]. Another example is complement C3, a key regulator of inflammatory responses, a molecule that is incorporated into the clot and modulates fibrinolytic potential (discussed below).

### Increased incorporation of anti-fibrinolytic proteins

A potentially important but overlooked diabetes-related mechanism for hypofibrinolysis is increased incorporation of antifibrinolytic proteins into the clots, specifically complement C3 and PI.

#### Complement C3

Until recently, complement C3 was regarded as an inflammatory protein, but evidence suggests key interactions between the complement and coagulation systems [102]. Two studies, using a proteomics approach, demonstrated the presence of C3 and its metabolites in plasma clots [103, 104]. C3 protein can be covalently crosslinked to fibrin by FXIIIa, as well as weakly associating with the clot via non-covalent interactions [105, 106].

C3 binding/cross-linking into the fibrin network increases resistance of the clot to lysis [104]. Further work from our laboratory has demonstrated that incorporation of C3 into the fibrin clots of T1DM subjects has a greater effect on prolongation of clot lysis compared with clots from healthy controls [107]. The association between elevated C3 plasma levels and prolonged clot lysis has also been observed in T2DM [108, 109]. In the study by Hess et al. of 875 patients with T2DM, a regression analysis involving PAI-1, fibrinogen, C3 and CRP plasma levels, demonstrated that C3 was an independent predictor of fibrinolysis potential in contrast to CRP which failed to show an independent association with clot lysis.

The mechanisms for C3-induced compromise in clot lysis are not entirely clear but one may involve C3 interference with tPA and/or plasminogen binding sites on the fibrin network. Alternatively, C3 may affect the availability of plasmin to act on fibrin fibres, as C3 is a known substrate for plasmin [110]. Finally, the presence of elevated levels of C3 in the fibrin clot may simply increase mechanical resistance of the clot to lysis [107]. From the therapeutic point of view, these findings suggest that targeted disruption of C3-fibrinogen interaction may offer a diabetes-specific anti-thrombotic treatment strategy.

#### Plasmin inhibitor (PI)

Plasmin inhibitor (α2-antiplasmin, α2-plasmin inhibitor) belongs to the serpin superfamily of proteins. PI is a key protein in blood haemostasis, as evidenced by the disorders caused by homozygous deficiency or by protein over-production. Congenital deficiency of PI results in a severe bleeding disorder [111, 112], whilst elevated levels of PI are associated with an increased risk of first MI [113]. PI is the main biological inhibitor of plasmin, free PI is able to bind plasmin and form an irreversible stable complex, however, the fibrin-bound form of PI seems to be the main regulator of clot lysis [114]. Cross-linking of PI to the fibrin clot by FXIIIa has been shown to be a key determinant of resistance to lysis [25].

Agren et al. found increased incorporation of PI into the fibrin network in individuals with T1DM, although these individuals exhibited a paradoxical reduction in clot lysis time, which the authors attributed to reduced PAI-1/fibrinogen levels [115]. An earlier study by Dunn et al. found increased cross-linking of PI into the fibrin networks in T2DM, which increased resistance to lysis compared with controls [65]. This provides yet another diabetes-specific therapeutic target by manipulating PI incorporation into fibrin networks.

### Compromised efficiency of the fibrinolytic system

A number of abnormalities in the fibrinolytic system have been reported in diabetes including elevated PAI-1 and TAFI levels, and deranged plasminogen function.

#### TAFI in diabetes

There is some evidence to suggest that antifibrinolytic protein TAFI is upregulated in diabetes, which could contribute to the hypofibrinolytic state. Elevated plasma levels of TAFI are associated with increased risk of cardiovascular diseases [116–120]. In diabetes, TAFI levels appear to correlate with HbA1c, indicating an association between TAFI levels and poor glycaemic control [121]. Plasma concentration and activity of TAFI are significantly increased in T2DM patients compared with healthy controls, particularly in obese patients [121]. TAFI levels have also been linked in T2DM to the presence of microvascular complications manifesting as microalbuminuria [122, 123]. However, Chudý et al. found no significant difference in TAFI levels when normoalbuminuric T2DM patients were compared with controls, and Yener and colleagues have demonstrated that TAFI levels do not increase in normotensive T2DM subjects without diabetic complications [123, 124]. To further complicate matters, others found TAFI levels to be decreased in nonobese T2DM individuals [125]. Taken together, these data reflect inconsistencies in the relationship between TAFI and diabetes, which is perhaps related to the heterogeneity observed in these patients. Therefore, studies including large numbers of diabetes patients are required, which would allow appropriate subgroup analysis, to fully understand the role of TAFI in diabetes.

#### PAI-1 in diabetes

PAI-1 levels are associated with increased risk of CVD [126] with elevated protein levels found in young (<45 years) survivors of myocardial infarction [127] and those with recurrent MI [128]. For many years, PAI-1, produced by endothelial cells and adipose tissue, has been regarded as the main inhibitor of fibrinolysis in diabetes. However, there is a difference between T1DM and T2DM as PAI-1 levels appear to be only elevated in the latter and correlate with glycaemic parameters as well as markers of insulin resistance [115, 129–133]. Hormonal (hyperinsulinemia) and metabolic (hyperglycemia and hypertriglyceridemia) derangements, typically found in T2DM patients, seem to have a role in elevated levels of PAI-1 levels in this population [130]. Stegenga et al. demonstrated that in healthy subjects, hyperinsulinemia inhibits fibrinolysis, primarily by enhancing PAI-1 secretion, whilst hyperglycaemia stimulates coagulation [134]. This group suggests that in T2DM subjects, the presence of both hyperinsulinemia and hyperglycaemia has a procoagulant effect with the simultaneous inhibition of fibrinolysis.

There is also an association of elevated PAI-1 levels and abdominal obesity as adipose tissue expresses PAI-1 and represents an important source of plasma PAI-1 in obese subjects [131–133]. This explains that the combination of T2DM and obesity contribute to an even greater elevation of PAI-1 than obesity or diabetes alone [135].

#### Plasminogen in diabetes

The hyperglycaemic environment in diabetes is responsible for glycation of plasminogen, which compromises protein function. Plasminogen purified from plasma of individuals with T1DM has a decreased rate of conversion to plasmin, and the plasmin generated has altered proteolytic activity, resulting in impaired fibrinolysis. Moreover, improving glycaemic control partially restores plasminogen conversion to plasmin and the activity of the enzyme, further emphasising the importance of maintaining good glycaemic control to reduce thrombosis risk. Interestingly, only a modest improvement in glycaemic control is enough to result in significant improvement in plasmin activity [136]. This suggests small improvements in glycaemic control translate clinically into significant reduction in thrombosis risk.

Therefore, hyperglycaemia enhances thrombosis potential by increasing plasma levels of pro-coagulant and antifibrinolytic factors as well as modulating protein activity by introducing post-translational modifications in coagulation proteins. It should be noted, however, that not every single factor that modulates fibrinolysis has been comprehensively studied in diabetes, particularly in relation to the various subgroups of patients. Therefore, in addition to developing agents that target the large number of anti-fibrinolytic proteins, future studies are required to understand the most relevant anti-fibrinolytic strategy in the different subgroups of diabetes patients. This may indeed involve targeting multiple proteins/pathways, particularly in the higher risk groups, to maximise clinical benefit.

**Fig. 3:**
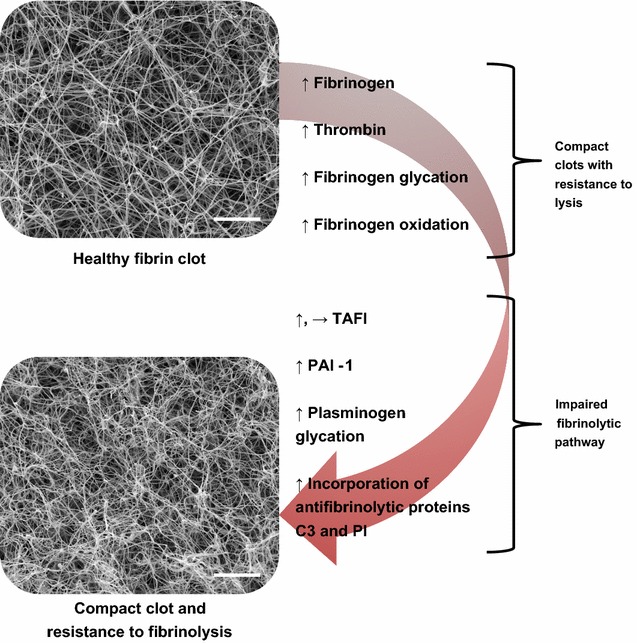
Mechanisms involved in hypofibrinolysis in diabetes. The main factors influencing hypofibrinolysis in diabetes include altered fibrin network structure and an impaired fibrinolytic system. Factors contributing to altered clot structure include elevated levels of thrombin, and both quantitative and qualitative alterations in fibrinogen, including glycation and oxidation of fibrinogen molecules. Elevated levels of plasminogen activator inhibitor (PAI-1), glycation of plasminogen and increased incorporation of antifibrinolytic proteins plasmin inhibitor (PI) and complement C3 into clots in individuals with diabetes all contribute to impaired fibrinolysis. Levels of thrombin activatable fibrinolysis inhibitor (TAFI) can be raised in diabetes but studies are conflicting with some showing no change. *Scale bar* 5 μm. ↑, increase; →, no change

A summary of the mechanisms involved in hypofibrinolysis in diabetes is provided in Fig. 3.

## Current approaches to reduce hypofibrinolysis in diabetes

The potential role of various hypoglycaemic agents in thrombosis risk is beyond the scope of this review and will therefore concentrate on the effect of glycaemia per se on thrombosis potential.

### Role of glycaemia

From the evidence presented above, it is clear that hyperglycaemia results in a prothrombotic and hypofibrinolytic environment. Moreover, relatively modest improvement in glycaemia appears to have a significant effect on fibrin network structure and/or resistance to lysis. It should be noted, however, that overtreatment of hyperglycaemia, and precipitation of hypoglycaemia, can also be prothrombotic [137]. Studies have shown that hypoglycaemia results in elevated fibrinogen and PAI-1 levels [138]. This is consistent with our findings of impaired fibrinolysis following hypoglycaemic clamps in diabetes individuals, with this enhanced prothrombotic milieu lasting for up to one week after the hypoglycaemic event [139]. The observation that both hyper and hypoglycaemia are prothrombotic adds another dimension to the management of this risk factor, particularly as glucose levels can fluctuate significantly in diabetes patients secondary to daily activities, diet and hypoglycaemic therapies.

Taken together, the above findings may offer mechanistic explanations for the disappointing clinical outcome trials investigating the role for tight glycaemic control in reduction of vascular ischaemic events in diabetes [140]. It is plausible that mild improvement in glucose is all that is needed to control the prothrombotic environment in diabetes. Trying to achieve too tight control runs the risk of repeated hypoglycaemia, predisposing to an enhanced thrombotic environment, thus negating any beneficial effect for reducing blood glucose levels.

From the practical point of view, using agents that are less likely to cause hypoglycaemia may have the advantage of reducing the thrombotic environment in diabetes and protecting against vascular ischaemic events. We have limited evidence to suggest that agents that do not cause hypoglycaemia, such as metformin, pioglitazone, empagliflozin and liraglutide are associated with favourable cardiovascular profile [141–144]. In contrast, agents that may result in hypoglycaemia such as sulphonylurea and insulin have been linked to increased cardiovascular risk [145]. However, there are complexities encountered in dissecting out the effect of each agent, given that most high risk individuals are on combination therapy, and therefore further research in this area is needed before concrete conclusions can be made.

### Potential diabetes–specific therapeutic targets to reduce hypofibrinolysis

Given that diabetes is associated with increased plasma levels of PAI-1 and TAFI, and increased incorporation of PI and C3 into the clot, targeting these proteins may alleviate the hypofibrinolytic environment, consequently decreasing atherothrombotic risk.

#### TAFI as a drug target

TAFI circulates in an inactive zymogen form and is activated by thrombin, plasmin, or the thrombin-thrombomodulin complex. Activated TAFI cleaves C-terminal lysine residues from partially degraded fibrin, which are critical for the binding of plasminogen and as a result, plasmin generation is reduced [146].

Inhibition of TAFI was considered as a therapeutic strategy in thrombotic disorders but only a limited number of drug candidates have made it to clinical trials, which were then discontinued (reviewed elsewhere [147, 148]). More recent work has investigated the TAFI-inhibitory ability of TAFI-derived peptides on the protein’s activation and activity. Peptides with the ability to prevent TAFI activation, and inhibit TAFIa activity directly were identified [149]. An alternative anti-TAFI approach by Buelens et al. created a panel of inhibitory nanobodies effective against the various modes of TAFI activation and activity. Nanobodies are single domain antibodies from the sera of members of the *Camilidae* family which have advantageous properties such as low immunogenicity and high affinity, solubility and stability [150]. Two nanobodies showed a potent profibrinolytic effect in an in vitro clot lysis assay and their interaction with TAFI was later characterised using X-ray crystallography. One nanobody was shown to bind close to the TAFI activation site, and the other close to a possible thrombomodulin binding site. These findings explained the interference of the two nanobodies with TAFI activation, and thrombin-thrombomodulin-mediated activation, respectively [151]. Although these studies are promising and shed light on the mechanistic properties of TAFI-inhibitory compounds, in vivo data are lacking.

#### PAI-1 as a drug target

PAI-1 is present in an active and a latent state in vivo with the active form having high affinity for vitronectin, which binds PAI-1 in the blood [152]. PAI-1 has a relatively short half-life of ~ 1 h in physiological conditions [153, 154] which some may argue would limit its use as a therapeutic target. The counter-argument, however, is that the short half-life makes it a suitable target for acute vascular thrombosis. Several PAI-1 inhibitors have been tested in vivo and in vitro and displayed inhibitory activity, but to date no PAI-1 inhibitor is clinically available. Prevention of cellular PAI-1 biosynthesis is another strategy which has been partially explored.

#### Small molecule inhibitors of PAI-1 synthesis

Small molecule inhibitors of PAI-1 synthesis include synthetic and natural products such as niacin, fibrates and butadiene derivatives (reviewed in [114]). However, these compounds are not PAI-1 specific, having other metabolic effects and therefore dissecting out their role on clinical outcome is difficult. Also, in vitro studies do not always translate into in vivo benefits and an example of the disparity can be seen with fibrate use; a recent meta-analysis has reported that fibrates do not cause a decrease in PAI-1 levels or activity, despite their in vitro effects [155]. Therefore, direct PAI-1 inhibitors may have an advantage by targeting plasma PAI-1.

#### Direct PAI-1 inhibitors

##### Antibodies against PAI-1

There have been numerous attempts at inhibiting PAI-1 with antibodies, reviewed elsewhere [148, 156] but none have been taken forward to the clinical arena. Recent work in mouse models of thrombotic stroke have shown that simultaneous inhibition of PAI-1 and TAFI with a bispecific antibody results in a significant enhancement of fibrinolysis in mice, without increased bleeding [157]. To dissect out the contribution of PAI-1 inhibition and TAFI inhibition during ischaemic stroke, Denorme et al. used monoclonal antibodies to identify the effect of inhibiting each of these antifibrinolytic factors. In a mouse model of transient middle cerebral artery occlusion, inhibition of TAFI or PAI-1 significantly decreased fibrin(ogen) deposition in the ischaemic brain, thereby improving reperfusion, but the combined inhibition had an additive beneficial effect [158]. PAI-1 inhibition is also possible with nanobodies, which was demonstrated by Zhou et al. who used nanobodies against PAI-1 to inhibit profibrinolytic activity in an in vitro clot lysis assay [159]. Clearly there are some promising benefits in the inhibition of PAI-1 with antibodies, which merit further in vitro and in vivo analysis.

##### Other PAI-1 inhibitors

A number of groups have employed different methods for identification of PAI-1 inhibitors, including high throughput and in silico screening, reviewed extensively elsewhere [114, 160]. Using this methodology, Elokdah and colleagues identified Tiplaxtinin as a potent and selective PAI-1 inhibitor [161], which became one of the most studied PAI-1 inhibitors to date. Tiplaxtinin binds specifically to the active conformation of PAI-1, its activity, however, is blocked by vitronectin, suggesting their binding sites on PAI-1 are overlapping [162]. In a canine model of occlusive thrombus formation, administration of Tiplaxtinin caused spontaneous coronary reperfusion, indicating active fibrinolysis. When Tiplaxtinin was administered to rats following induction of an occlusive arterial thrombosis, thrombus weight was reduced and blood flow facilitated with increased occlusion time in the affected vessel. In both these models, there were no side effects reported such as changes in heart rate, blood pressure or increased bleeding [161].

Other small molecule inhibitors have been identified, such as ZK4044 which binds directly to PAI-1, preventing its interaction with target proteases [163], WAY-140312 which binds and inactivates PAI-1 [164], and S35225 a benzothiophene derivative which, in contrast to WAY-140312 and Tiplaxtinin is able to inhibit PAI-1 in the presence of vitronectin [165]. Despite promising candidates, there has been no progression of PAI-1 inhibitors to clinical trials in man [166]. One explanation for this lack of progress could be the unique structural properties of PAI-1. Crystallographic data of PAI-1 in complex with vitronectin is still incomplete and thus hampers rational design of small molecules able to bind and inactivate PAI-1 in its vitronectin-bound state. Additionally, the active form of PAI-1 is yet to be crystallised due to its instability, and the mechanism by which active PAI-1 transitions to its latent form is not fully understood. Although numerous PAI-1 inhibitory compounds have been investigated, for many, their mechanism of action remains elusive, providing yet another obstacle in the clinical application of PAI-1 inhibitors.

#### Plasmin inhibitor as a drug target

The reported increased incorporation of PI into fibrin clots in diabetes renders this protein a potential diabetes-specific therapeutic target. Numerous attempts at targeting PI have been made, including the use of antibodies and mutant forms of the protein.

#### Antibodies

Kumada et al. reported that repeated injection of polyclonal anti-PI F(ab’)_2_ fragments reduced circulating PI levels and led to an acceleration of thrombolysis by enhancing fibrinolytic activity [167]. A more targeted approach by Sakata et al. saw the creation of a monoclonal antibody that was able to interfere with the formation of PI-plasmin complexes by recognising an epitope either in or close to the reactive site of PI. This antibody, termed JTPI-1, was able to increase the effectiveness of tPA-mediated clot lysis in plasma [168]. Similarly, an antibody capable of inhibiting both PI in plasma and clot-bound PI caused spontaneous clot lysis and also enhanced tPA-mediated clot lysis in vitro [169]. An extension of these studies in vivo by Reed et al. used an antibody targeted to clot-bound PI, which was found to enhance lysis of a human clot in a rabbit jugular vein thrombosis model [170].

#### Plasmin inhibitor mutants

Arginine residue 364 is the main reactive site on PI, which forms a covalent bond with the active site serine in plasmin, resulting in an inactive protease-inhibitor complex [171, 172]. Lee et al. generated PI with a chemically modified reactive site arginine [173] and an Arg-Ala mutant [174]. Both of these modified PI molecules lost their plasmin-inhibitory activity, but still competed with native PI for crosslinking into the fibrin clot by FXIIIa, thereby enhancing fibrinolysis.

#### N and C terminal peptides of plasmin inhibitor

The N-terminal region of PI is crucial for cross-linking to fibrinogen [175, 176], whereas the C-terminal of the protein mediates interaction with plasmin [177]. Kimura et al. created a 12 residue synthetic N-terminal peptide of PI that was able to reduce incorporation of native PI into fibrin networks by FXIIIa in vitro. Cross-linking of the synthetic peptide to fibrin accelerated spontaneous as well as tPA-induced fibrinolysis. The quantity of native PI cross-linked to fibrin was proportionally reduced by the presence of the synthetic N-terminal peptide, indicating PI specificity of the synthetic peptide [178].

Others attempted to use the C-terminus of PI as a means of enhancing fibrinolysis. A 26 amino acid peptide composed of the carboxy terminal region of PI was found to enhance in vitro fibrin clot lysis by increasing conversion of plasminogen to plasmin approximately fivefold [179]. Similarly, Udvardy et al. observed that a peptide containing the 26 amino acid C-terminal of PI, coupled with RGD (Arg-Gly-Asp) peptide was capable of simultaneously promoting fibrinolysis and inhibiting platelet aggregation. Fibrinogen RGD sequence mediates its interaction with platelets, RGD peptides are able to disrupt fibrinogen-platelet interactions and have platelet inhibitory effects, as observed in this study [180].

#### Inhibition of plasmin inhibitor cleavage

Plasmin inhibitor is present in two forms in human plasma, a 464-residue protein with methionine at the N-terminus (Met-PI) and a truncated 452-residue protein with asparagine at its N-terminus (Asn-PI). Human plasma contains around 30% Met-PI and 70% Asn-PI form. Asn-PI, formed from Met-PI by the action of antiplasmin cleaving enzyme (APCE), is more rapidly cross-linked to fibrin by FXIIIa [181–184]. APCE represents a therapeutic target, since inhibition of this enzyme could reduce circulating levels of Asn-PI and therefore decrease incorporation of PI into the fibrin clot. Indeed, Lee et al. observed an enhancement of plasma clot lysis when APCE was inhibited in vitro [185] but in vivo studies are lacking.

Despite promising preliminary data, none of these approaches of PI-related therapeutic targets have been clinically adopted, indicating that translating in vitro findings, and even animal work, into application in man is difficult and more complex than initially envisaged.

#### Complement C3 as a drug target

Therapeutic intervention in the complement system has been recognised as a potential strategy for the treatment of a series of inflammatory and autoimmune diseases [186, 187] whilst the role of C3 in fibrinolysis has yet to be widely recognised as an antithrombotic therapeutic target. Early work has shown that one approach in modulating the antifibrinolytic action of C3 has involved using a phage display system of small conformational peptides termed Adhirons. One Adhiron was identified which abolished C3-induced prolongation of fibrin clot lysis by interfering with the C3-fibrinogen interaction. Importantly, this Adhiron, being specific for fibrinogen potentially avoids targeting of C3 systemically, narrowing the therapeutic window and lessening the risk of off-target effects [188]. It remains to be seen whether this approach proves to be suitable for future clinical application.

Potential future strategies to enhance the fibrinolytic process, and reduce thrombosis risk in diabetes are summarised in Table 2.

**Table 2:** Current and potential future strategies to enhance fibrinolysis and reduce thrombosis risk in diabetes

Modulatory mechanism	Current/potential therapeutic strategies
Improve glycaemic control	Agents which control blood glucose while avoiding hypoglycaemia
Decrease levels of active TAFI	Inhibit activation of TAFI [147–151] Direct TAFIa inhibitors [147–151]
Decrease levels of active PAI-1	Inhibition of PAI-1 synthesis [114] Direct PAI-1 inhibition [114, 148, 156–165]
Decrease incorporation of plasmin inhibitor (PI) into the clot	Antibodies [167–170] PI mutants [173, 174] N- and C-terminal peptides of PI [178–180] Inhibition of antiplasmin cleaving enzyme [185]
Decrease incorporation of C3 into the clot	Targeted disruption of the fibrinogen-C3 interaction [188]

## Conclusions/summary

Despite advances in treatment, vascular mortality in patients with diabetes remains unacceptably high, which is partly related to the enhanced thrombotic environment that is not fully controlled with current anti-platelet therapies. Rather than developing more effective anti-platelet agents, which runs the risk of bleeding complications, an alternative approach is to target the hypofibrinolytic state in diabetes, a key abnormality in this condition. Therefore, understanding the mechanisms for increased fibrin-related thrombosis risk in diabetes is key to develop effective and safe novel antithrombotic therapies.

Diabetes is associated with both quantitative and qualitative changes in clotting factors resulting in denser fibrin networks that are more difficult to lyse. In addition to changes in clot structure, diabetes directly impairs the function of the fibrinolytic system and shows increased incorporation of anti-fibrinolytic proteins into the clot. More specifically, recent studies have shown increased incorporation of PI and complement C3 into diabetes clots, which compromise fibrin clot lysis. The increased incorporation of anti-fibrinolytic proteins into fibrin networks represents a novel diabetes-specific mechanistic pathway that may be a target for a new generation of anti-thrombotic agents.

In summary, targeting alternative and diabetes-specific thrombotic pathways may be the best approach to reduce the residual thrombosis risk in diabetes and improve vascular outcome in this population.
